# Soft Tissue Sarcomas: Treatment and Management

**DOI:** 10.3390/cancers16051042

**Published:** 2024-03-04

**Authors:** Shinji Miwa, Po-Kuei Wu, Hiroyuki Tsuchiya

**Affiliations:** 1 Department of Orthopedic Surgery, Graduate School of Medical Sciences, Kanazawa University, Kanazawa 920-8640, Japan; tsuchi@med.kanazawa-u.ac.jp; 2 Department of Orthopedics and Joint Reconstruction, Taipei Veterans General Hospital, Taipei 112, Taiwan; drwuvgh@gmail.com; 3 Orthopedic Department of Medicine, National Yang Ming Chiao Tung University, Taipei 112, Taiwan; 4 Therapeutical and Research Center of Musculoskeletal Tumor, Taipei Veterans General Hospital, Taipei 112, Taiwan

## 1. Introduction

Due to the rarity and heterogeneity of soft tissue sarcoma (STS), investigating new treatments for this condition has been challenging. Although intensive chemotherapy and the establishment of surgical procedures have improved the outcome of patients with STS, there are ongoing issues such as limited anticancer agents, a high incidence of postoperative complications, and an unsatisfactory curative rate for recurrent/metastatic STS. To improve the clinical outcomes of patients with STS, there is a need for further investigations into molecular biology, the microenvironment, anticancer agents, and the management of STS. Thus, this Special Issue collates various high-quality original/review articles on basic and clinical research into STS.

## 2. An Overview of Published Articles

This Special Issue comprises both original and review articles focusing on systemic treatment and complications in STS management. Grünwald et al. conducted a prospective phase 4 study assessing the use of trabectedin in 128 patients with STS [contribution 1]. The progression-free survival rates at 3 and 6 months were 61% and 45%, respectively. Of the study participants, 1 patient had a complete response, 14 had a partial response, and the objective response rate was 12%. Common grade 3/4 adverse events included leukopenia (27%), thrombocytopenia (16%), neutropenia (13%), and increased alanine aminotransferase (11%). Two patients died as a result of sepsis and pneumonia, and these were deemed to be treatment-related adverse events. Hoshi et al. investigated the influence of systemic chemotherapy on skeletal growth in 20 patients (aged ≤18 years) with osteosarcoma [contribution 2]. According to their findings, systemic chemotherapy did not inhibit skeletal growth in young patients with osteosarcoma. Fazel et al. summarized the state of the art of immunotherapy in STS in their review article [contribution 3]. Recent clinical studies on immune checkpoint inhibitors suggest that the efficacy of this treatment was limited, necessitating further studies on combination therapies, innovative adoptive therapies, and biomarkers. To assess global health-related quality of life during treatment with pazopanib or physician-preferred chemotherapy over a 9-week period, a prospective, randomized, controlled, multicenter study (PazoQoL) was designed [contribution 4]. Although the study was discontinued due to the pandemic, continuous electronic patient-reported outcomes enabled early detection of the onset of deterioration and initiation of countermeasures. The Time Trade-off demonstrated that the prolongation of life and the side effect profile of continued therapy failed to meet patients’ expectations.

This Special Issue also includes three articles on gastrointestinal stromal tumors (GISTs). Management of small gastrointestinal stromal tumors (GISTs), which are less than 2 cm in size, remains controversial. Guo et al. reported a high overall mutation rate (96%) and high mutation rate of oncogenic BRAF-V600E in small GISTs [contribution 5]. Although previous studies showed a significantly lower mutation rate of small GISTs compared with large tumors [1,2,3], these results indicate that genetic alterations are common in early GIST generation. Alfagih et al. presented pathological data, management strategies, and clinical outcomes of 248 patients diagnosed with GISTs in a single cancer center [contribution 6]. At diagnosis, 206 patients (83%) had a localized tumor. A total of 213 patients (86%) underwent curative surgical resection, while 49 patients (20%) underwent adjuvant imatinib. The 5-year overall survival rates of patients with low-, intermediate-, high-risk, and advanced tumors were 100%, 94%, 91%, 88%, and 65%, respectively. Univariate analysis revealed that the location of the tumor, Eastern Cooperative Oncology Group performance status, secondary malignancy, size, and mitosis were predictors for overall survival. GISTs are believed to be resistant to radiation therapy [4]. On the other hand, objective responses to radiation therapy have been described in case reports and cases series [5,6]. To assess the role of radiation therapy in GISTs, Zhang et al. conducted a systematic review of radiation therapy for the treatment of GISTs [contribution 7]. In the review, bone was the site most commonly treated with radiation therapy. In the review, radiation therapy showed objective response in some patients with advanced or metastatic GISTs, although no survival benefit was observed. The symptom palliation rate was 79%, and radiation therapy was generally well tolerated. Further studies on radiation therapy for GISTs are needed to identify the indication of radiation therapy for GIST.

Although radiation therapy is thought to be an effective treatment option in patients with high-grade sarcoma, positive margin, or unresectable sarcomas, this type of therapy increases wound complications. Seidensaal et al. conducted a prospective, one-armed, single-center phase 1/2 study on preoperative dose-escalated intensity-modulated radiotherapy (IMRT) and intraoperative radiation therapy (IORT) in patients with retroperitoneal STS [contribution 8]. In their study, 37 patients underwent preoperative IMRT of 45–50 Gy with a simultaneous integrated boost of 50–56 Gy, surgery, and IORT. Twenty-seven participants underwent IORT, thirty-five patients underwent tumor resection, and the surgical margin was positive in twenty-eight patients (80%) and negative in seven patients (20%). The 5-year overall survival rate was 60%. The authors described that stratification by grading and histology should be considered for future studies. Radiation-induced fibrosis, a severe side effect of radiation therapy, is induced by TGF-β1, Smad2/3 phosphorylation, and profibrotic target genes [7,8]. Cruz-Morande et al. reported that P144, a TGF-β1 peptide inhibitor, reduced radiation-induced fibrosis and significantly reduced Smad2/3 phosphorylation [contribution 9]. Further studies on the optimal dosage and timing of P144 administration in model of radiation-induced fibrosis are required. Preoperative radiation therapy increases the risk of postoperative wound complication in the treatment of STS. Ouyang et al. conducted a retrospective study using the Oxford University Hospital database [contribution 10], which included 126 patients with STS who underwent preoperative radiation therapy. In multivariate analysis, age, tumor size, and metastasis were identified as independent risk factors for major wound complication. They also used a nomogram, a useful tool that enables clinicians to assess the risk of major wound complication by graphical calculation of each predictor, and it showed a good predictive value for major wound complications in this study.

This Special Issue also includes several studies on the management of metastatic leiomyosarcomas. Delisle et al. conducted a systematic review and pooled analysis to investigate survival in patients who underwent metastasectomy for leiomyosarcoma and to compare their outcomes based on the site of metastasis [contribution 11]. The median survival rates were 73 months for lung metastasectomy, 35 months for liver metastasectomy, 14 months for spine metastasectomy, and 14 months for brain metastasectomy. Two studies comparing outcomes between patients revealed that metastasectomy was associated with significantly better survival rates. Although these studies were nonrandomized, these data suggest that metastasectomy offers numerous clinical benefits. Imura et al. investigated the clinical features, outcomes, and prognostic factors of metastatic extrauterine leiomyosarcoma [contribution 12]. Sixty-one patients with metastatic extrauterine leiomyosarcoma were included in the retrospective study. The five-year overall survival of the study patients was 38%. Univariate analysis showed that primary tumor size (>10 cm), initial metastatic sites >1, synchronous metastasis, and no metastasectomy were significantly associated with poor overall survival. In multivariate analysis, primary tumor size was identified as an independent prognostic factor for poor overall survival. Among 24 patients who underwent metastasectomy, the interval from the initial diagnosis to development of metastasis (≤6 months) was significantly associated with poor overall survival. In 37 patients without metastasectomy, chemotherapy was significantly associated with better overall survival. This study suggested that metastasectomy and chemotherapy could improve overall survival in patients with metastatic extrauterine leiomyosarcoma.

Due to the rarity of angiosarcoma of the breast, the optimal treatment method remains unclear. Kim et al. reported clinicopathological features, treatment, and oncological outcomes of primary angiosarcomas of the breast [contribution 13]. In their study, 15 patients with primary angiosarcoma of the breast underwent surgical tumor resection. The mean age of the patients was 33 years and mean tumor size was 7.7 cm. The histological grades were low-grade in three cases, intermediate-grade in five cases, high-grade in six cases, and unidentified-grade in one case. The 5-year disease-free and overall survival rates were 24% and 37%, respectively. Histological grade was significantly associated with overall survival (*p* = 0.024). Because the roles of chemotherapy and radiotherapy remain unclear, surgical resection with appropriate surgical margin is thought to be the best approach to treating angiosarcoma of the breast. Mangla et al. investigated the risk of mortality and prognostic factors for primary hepatic angiosarcoma using the National Cancer Database (NCDB) [contribution 14]. In their study, 346 patients with primary hepatic angiosarcoma were included. The mean age of the patients was 63 years. One-third of the patients (36%) underwent chemotherapy, while 15% underwent surgical tumor resection. The survival rates in patients with surgical tumor resection and those without surgical resection were 8 and 2 months, respectively (*p* < 0.001). Patients who underwent chemotherapy had significantly better survival than patients without chemotherapy (5 months vs. 1 month, *p* < 0.001), although no long-term survival benefit was observed.

Kito et al. conducted a retrospective multicenter study that explored the clinical features and prognosis of low-grade myofibroblastic sarcoma [contribution 15]. The study included 24 patients with low-grade myofibroblastic sarcoma; of these, 22 patients underwent surgical tumor resection and 2 patients underwent radiation therapy. The status of the surgical margin was R0 in 14 cases, R1 in 7 cases, and R2 in 1 case. The local recurrence-free survival was 91% at 2 years and 75% at 5 years. The two patients who underwent radiation therapy showed one complete response and one partial response. This study suggests that tumor resection with appropriate surgical margin is considered to be the standard treatment, and radiation therapy is an alternative treatment option in unresectable low-grade myofibroblastic sarcoma.

MicroRNAs (miRNAs), short non-coding RNA molecules, target and modulate various dysregulated genes and/or signaling pathways in cancer cells. miRNAs are believed have utility in the diagnosis, prognosis prediction, and treatment of STS. In their review article, Teo et al. provided an updated discussion of roles and potential use of miRNAs in management of STS [contribution 16]. 

A computer-generated 3D tumor model can reveal tumor and adjacent neurovascular structures. Fang et al. reported that using a computer-generated 3D tumor model of axillary STS reduced intraoperative blood loss, operative time, and hospital stay [contribution 17]. This technique can be expected to improve operative planning in patients with STS adjacent to important organs. 

Cao et al. developed a theoretical model for thermal nonlinear photoacoustic detection related to port-wine stain samples [contribution 18]. 

Tamura et al. reported a case of pleomorphic rhabdomyosarcoma during the follow-up of a uterine leiomyoma [contribution 19].

In summary, this Special Issue presents a collection of articles that discuss the latest basic and clinical studies on STS. Although there are limited treatment options for patients with STS, recent studies have demonstrated promising therapeutic targets, anticancer agents, and combinations of these treatments. We hope the articles presented in this Special Issue will help improve the management of STS. We greatly appreciate the contributions of the authors of the articles published in this Special Issue.
